# Neural Modulation of the Primary Auditory Cortex by Intracortical Microstimulation with a Bio-Inspired Electronic System

**DOI:** 10.3390/bioengineering7010023

**Published:** 2020-03-02

**Authors:** Maria Giovanna Bianco, Salvatore Andrea Pullano, Rita Citraro, Emilio Russo, Giovambattista De Sarro, Etienne de Villers Sidani, Antonino S. Fiorillo

**Affiliations:** 1 Department of Health Sciences, Magna Græcia University of Catanzaro, 88100 Catanzaro, Italy; citraro@unicz.it (R.C.); erusso@unicz.it (E.R.); desarro@unicz.it (G.D.S.); nino@unicz.it (A.S.F.); 2 Montreal Neurological Institute, McGill University, Montreal, QC H3A 2B4, Canada; etienne.de-villers-sidani@mcgill.ca

**Keywords:** sensor system, piezoelectric transducers, ECoG recording, neuromodulation, neuroprosthetics

## Abstract

Nowadays, the majority of the progress in the development of implantable neuroprostheses has been achieved by improving the knowledge of brain functions so as to restore sensorial impairments. Intracortical microstimulation (ICMS) is a widely used technique to investigate site-specific cortical responses to electrical stimuli. Herein, we investigated the neural modulation induced in the primary auditory cortex (A1) by an acousto-electric transduction of ultrasonic signals using a bio-inspired intracortical microstimulator. The developed electronic system emulates the transduction of ultrasound signals in the cochlea, providing bio-inspired electrical stimuli. Firstly, we identified the receptive fields in the primary auditory cortex devoted to encoding ultrasonic waves at different frequencies, mapping each area with neurophysiological patterns. Subsequently, the activity elicited by bio-inspired ICMS in the previously identified areas, bypassing the sense organ, was investigated. The observed evoked response by microstimulation resulted as highly specific to the stimuli, and the spatiotemporal dynamics of neural oscillatory activity in the alpha, beta, and gamma waves were related to the stimuli preferred by the neurons at the stimulated site. The alpha waves modulated cortical excitability only during the activation of the specific tonotopic neuronal populations, inhibiting neural responses in unrelated areas. Greater neuronal activity in the posterior area of A1 was observed in the beta band, whereas a gamma rhythm was induced in the anterior A1. The results evidence that the proposed bio-inspired acousto-electric ICMS triggers high-frequency oscillations, encoding information about the stimulation sites and involving a large-scale integration in the brain.

## 1. Introduction

In recent years, neuroprostheses have shown great potential in the investigation of the dynamic circuits of the brain due to the advances in biomedical engineering, [1,2,3]. Neuroprostheses are commonly categorized according to the brain areas involved in motor, sensory, sensorimotor, and cognitive functions [4]. In particular, the aim of sensory prostheses is to replace/restore lost sensory functions, allowing an exchange of information with the external environment. For instance, cochlear implants have allowed more than 100,000 people to compensate their impairments in perceiving speech [5], and retinal prostheses elicit visual percepts [6]. Besides peripheral prosthetic devices, another area of intense research effort is represented by implantable devices based on electrical stimulation of the neural cortex (i.e., intracortical microstimulation (ICMS)) to reactivate sensory responses [7,8]. In animal models, it has been shown that electrical stimulation of the sensory cortex causes electrically evoked percepts correlated to incoming stimuli exploiting the adaptability of the neocortex [4,5].

In Chapin’s [9] study, rats were guided to move in an environment as a robot by an experimenter, adopting microstimulation of the somatosensory cortex. Brief trains of stimulus pulses were delivered, training rats for navigation through three-dimensional structures [9]. Indeed, there is extensive literature available on brain plasticity that focuses on the ability of neuroprosthetic devices to manipulate native sensorial perception by providing external artificial stimuli. Thomson et al. [10] investigated the effects of infrared light neural devices implanted into the primary somatosensory cortices of lab rats. Their neuroprosthesis showed that the rats’ brains could perceive light from infrared (IR) sources, since the head-mounted sensors converted this light into ICMS, acting directly on receptive cortical areas. This device could be used to replace or augment the animals’ natural vision. Norimoto and Ikegaya [11] manipulated primordial sensory transduction using geomagnetic signals. In response to intracortical microstimulation of the primary visual cortices, blind rats learned to move about in mazes similarly to sighted rodents, adopting navigation strategies in response to stimulation received via their head-mounted microstimulators [11]. In such neuroprosthetic devices, the common factor is represented by the electrical microstimulator that directly affects the recruitment of neuronal population through direct depolarization of synaptic terminals or by action potentials close to the stimulation electrode [12]. Neurons are sensitive to the incoming stimuli and, after reaching the spike threshold, the response is amplified and synchronous with the input. The direct effect on neurons of specific regions of brain represents a distinct type of sensory, motor, or cognitive information that can be used in sensorial perception [12].

In light of these findings, we investigated an electronic interface, inspired by mammals’ echolocation system, for delivering sensory information through intracortical microstimulation. Since electrical stimuli can elicit a percept, our primary goal was to evaluate the effect of intracortical microstimulation generated by an ultrasonic source, namely, from a biomimetic sonar system, properly converted into electrical stimuli [13,14].

The system included an ultrasonic (US) transmitter and receiver made of a thin sheet of polyvinylidene fluoride (PVDF) assembled in a recently developed spiral-shaped geometry [15]. In addition, an electronic interface for the generation, conditioning, and processing of ultrasonic signals was also designed and fabricated. Each circuit was directly inspired by natural sonar observation, simulating the inner ear of the mammalian, in which acoustic waves were electrically transduced in electrical signals. Why ultrasounds as a source of information? Rodents adopt ultrasound vocalizations (USVs) at 22 kHz and 50 kHz for communication among conspecifics in aversive and alarming function or appetitive situations, respectively, and Microchiroptera use echoes for echolocation [16,17,18,19,20,21]. Firstly, we investigated the sensory processing that characterizes the acoustic transduction from ultrasonic waves into neural signals, from the cochlea up to the primary auditory cortex (A1). This area was mapped for ultrasound acoustic stimuli at 22 kHz and 50 kHz USVs [18,19,20,21]. Experimental results allowed the identification of potential stimulation sites through the evaluation of the tonotopic gradient of high-to-low best frequencies (BFs) oriented in the anterior-to-posterior direction in the A1. Subsequently, in order to bypass the peripherical pathways, ICMS stimulation generated by reproductive ultrasonic vocalizations and transduced into electrical stimuli was delivered to the A1.

Electrocorticography (ECoG) recordings showed an increase in amplitude modulation following microstimulation, evidencing either activation or inhibition of both the anterior and posterior areas. Power spectral density (PSD) analysis showed that oscillations in the alpha and beta bands in the posterior field of the A1 are highly specific for 22 kHz signals, whereas alpha and gamma oscillations were observed in the anterior areas of the A1 by stimulation with 50 kHz echoes. 

The ultrasound-based electric stimulation triggers high-frequency oscillations, encoding information about the stimulation sites and involving a large-scale integration in the brain.

## 2. Materials and Methods 

The first step of the experimental procedure consisted of the evaluation of the A1 receptive fields related to acoustic stimulation with 22 kHz and 50 kHz USVs. Recorded multi-unit activity through a 64 channel microelectrode array was post-processed, and the analysis of the neural activity in response to USV stimuli was evaluated. Subsequently, an intracortical stimulation was performed on the receptive fields previously mapped to verify the neural effects on the fronto-parietal cortex. The electrical stimuli were generated emulating the ultrasonic calls naturally used in ethological communication among rats.

### 2.1. Mapping of the Primary Auditory Cortex

#### 2.1.1. Animal Preparation and Neural Activity Recording 

**Figure 1:**
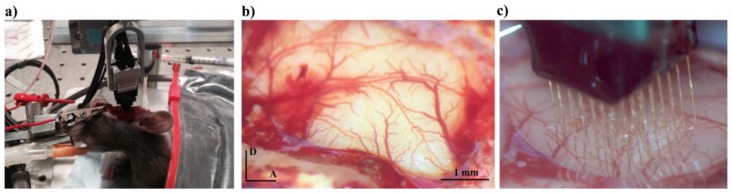
In vivo image of the mapping of the primary auditory cortex, A1 (**a**), delimited by local blood vessels (**b**) and the implanted 8 × 8 microelectrodes array (**c**). “A” stands for anterior direction, and “D” for dorsal direction.

The cortical mapping of the A1 was carried out at McGill University (Montreal Neurological Institute). All experimental procedures were approved by the Montreal Neurological Institute Animal Care Committee, and the guidelines of the Canadian Council on Animal Care were followed. Eight adult Long–Evans rats (22–24 months, 400–600 g) were anesthetized using ketamine/xylazine/acepromazine (65/13/1.5 mg/kg, intraperitoneal, i.p.), followed by a continuous delivery of isoflurane (1% in O_2_) after receiving buprenorphine (0.05 mg/kg) and carprofen (5 mg/kg) for analgesia. Body temperature was monitored with a rectal probe and maintained at approximately 37 °C by means of a homeothermic blanket system. Stereotaxic survival surgeries were performed using a custom-designed head holder, holding the rat by the orbits, leaving the ears unobstructed and, thus, allowing for sound stimulation during the surgery. Vital signs were monitored until full ambulation and recovery of stable hearing. At the end of the experimental test, rats were euthanized with an anesthetic overdose of pentobarbital (80 mg/kg). A craniotomy was performed to expose the auditory cortex and the left temporalis muscle was reflected as shown in Figure 1.

**Figure 2:**
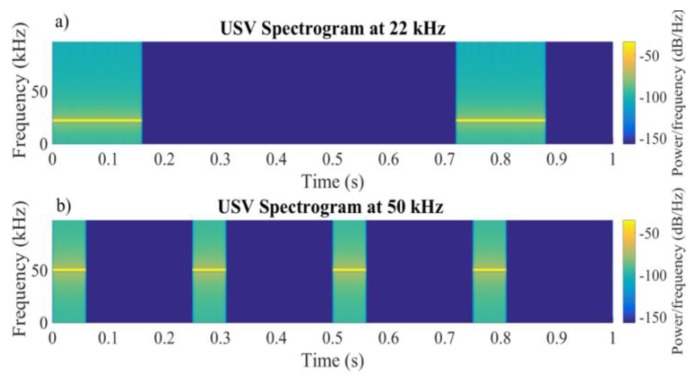
Ultrasound vocalizations (USVs) at 22 kHz with an overall duration of 7.92 s (each one consisted of 11 calls of 0.16 s) (**a**) and at 50 kHz with a duration of 6.74 s (26 calls of 0.06 s) (**b**). The vocalizations were presented to the rats 20 times.

An array of 8 × 8 tungsten microelectrodes (Tucker–Davis Technologies, Alachua, FL, USA) was implanted orthogonally into the primary auditory cortex at a depth of 500–650 μm (layers 4/5) using a hydraulic microdrive stereotaxic system (FHC, Bowdoinham, ME, USA) and fixed with dental acrylic and dental cement. Acoustic stimuli at ultrasonic frequency were generated using the TDT System III (Tucker–Davis Technology, Alachua, FL, USA) and delivered in a free field manner to the left ear through a calibrated speaker (TDT). The USVs were then presented to rats in a sound-attenuated chamber by way of a magnetic speaker (MF–1, Tucker-Davis Technologies), and the cortical responses were recorded using OpenEx and RZ6 auditory processing hardware (Tucker–Davis Technology, Alachua, FL, USA). Artificial USVs were designed using MATLAB, and each vocalization consisted of 11 calls at 22 kHz and 26 calls at 50 kHz, as detailed in Figure 2, according to natural recordings reported in Reference [21]. 

#### 2.1.2. Data Analysis

**Figure 3:**
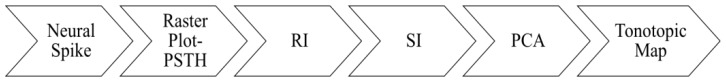
Flow chart on data processing for identification of A1 receptive fields. PSTH, peri-stimulus time histogram; RI, response index; SI, sensitivity index; PCA, principal component analysis.

The recorded multi-unit activity (MUA) was amplified (10,000×), filtered (0.3–5 kHz), and monitored on-line. Multi-unit spikes were detected with an automated algorithm using principal component analysis (PCA) (OpenSorter; Tucker–Davis Technology, Alachua, FL, USA). Neuronal features were investigated through the analysis of neural spikes in response to USV stimuli. Figure 3 illustrates the data processing and statistical analysis workflow for the classification of the A1 receptive fields. 

The recording channels were discarded if they were classified as belonging to the anterior auditory field (AAF). Neural activation patterns were analyzed by means of raster plots made of each spike and a sorting based on the response strength of the first stimulus. Peri-stimulus time histograms (PSTHs) were elaborated from the raster plots, averaging the neural responses, in order to visualize the temporal events evoked by the acoustic stimuli. Responses were estimated as the total number of spikes elicited after 20 presentations of the stimuli. The response map of the A1 neurons represents the cortical population having the highest discharge rate at the best frequency. It was found that the firing rate increased when the ultrasonic spectral content matched the best frequency of the receptive field. A response index (*RI*) was evaluated as the mean difference of each spike to the USV calls and to the corresponding baseline. Therefore, for the 64 channel microelectrode array, it follows that:

(1) $$RI=\bar{\overset{1280}{\underset{k=0}{\sum }}\left(x_{k}-B_{k}\right)}$$

where *B_k_* and *x_k_* are the baseline level and the vector containing the counts, respectively, as related to the *k*th trials of each channel. A Voronoi tessellation map provided a graphical representation of the organization of the A1 based on the *RI* [22]. The sensitivity of the specific subareas to the tonotopic frequency was evaluated using the sensitivity index (*SI*):

(2) $$SI=\frac{RI_{22kHz}-RI_{50kHz}}{2}$$

The PCA, applied to the recordings of MUA with 8 × 8 channels, was used to cross-validate the map obtained by the *RI* and *SI* calculations [23]. Spike data acquired from an electrophysiological recording system were converted into a matrix with each row constituting a variable, permitting the calculation of a covariance matrix. The covariance mappings succeeded in classifying groups, thus validating the previous analyses.

### 2.2. Electrical Stimulation with a Biomimetic Sonar System

#### 2.2.1. The Biomimetic Sonar Interface

**Figure 4:**
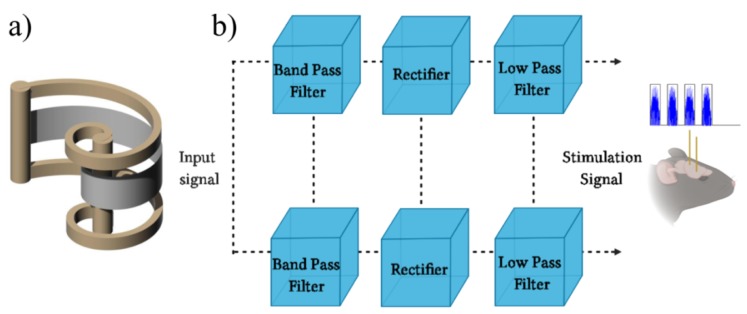
Biomimetic sonar system in reception of 22 kHz and 50 kHz echoes that are transduced in electrical unipolar stimuli. The functioning is based on two spiral-shaped ultrasonic transducers for generating and receiving wideband signals (**a**). Once the ultrasonic signal is received, it is processed through a bench of band-pass filters, precision rectifiers, and low-pass filters in order to obtain signals like the bioelectric patterns that normally travel along the acoustic nerve (**b**).

The high performance of mammalian biosonar makes it attractive to emulate, both from a biological and a technical point of view. A bio-inspired model, which behaves very similarly to the peripheral auditory system, in particular, from its external ear to the cerebral cortex, (i.e., rats and bats), was elaborated. Artificial USVs were generated by a polyvinylidene fluoride (PVDF)-based sonar system [24], because PVDF films are shaped according to appropriate geometries and can be used individually or in a wide variety of matrix configurations to create light, non-bulky sensory systems [25,26]. Recently, a novel spiral-shaped geometry was investigated to overcome the frequency band and directivity limitations, achieving uniform sensitivity on both the horizontal and vertical planes as a receiver and omni-directionality as an ultrasonic transmitter in the range 30–95 kHz [15,27]. The sonar was composed of two spiral-shaped unimodal transducers: one transmitter and one receiver (Figure 4a). Both were fabricated by folding a PVDF film (80 × 5 mm) uniaxially stretched along direction 1 and with a thickness of 28 μm; they were metalized on both sides through the vacuum deposition of a 200 nm thick layer of aluminum. A logarithmic spiral shape was imposed on the transducers, allowing the possibility of working in a wideband [15]. The acoustic wave was generated and received by a couple of wideband, spiral-shaped transducers (Figure 4a). Thus, as happens in the auditory nuclei following the cochlea, there was a vector of neurons tuned to different frequencies. This operation was performed by a bank of band-pass filters, each of which allows to select the desired frequency component (i.e., second-order active Sallen-Key (KRC) filters). Since the output of the filters was still far from being considered a bio-impulse, the sonar transmission, segmented into overlapping bands, was rectified with special double-wave precision rectifiers (i.e., the 1N34A-superdiode precision rectifier). Finally, since these bands still retained their pulsating nature, they were filtered with a bank of low-pass filters so as to obtain the envelope (see Figure 4b). The whole electronic system and the anesthetized mammals were placed in separate anechoic chambers in order to avoid the direct perception of ultrasounds.

#### 2.2.2. Electrical Stimulation

**Figure 5:**
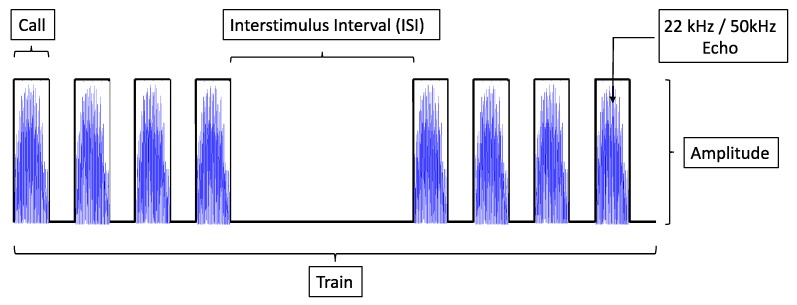
Design of electrical stimulation parameters; each call contains the 22 kHz/50 kHz rectified echoes, and it is repeated 11 or 26 times according to the USV type (11 calls of 0.16 s for USV at 22 kHz and 26 calls of 0.06 s for USV at 50 kHz).

The unipolar signals used for stimulating the A1 areas were generated mimicking the natural ultrasound vocalizations at 22 kHz and 50 kHz and transduced in electrical stimuli. Each impulse contained the rectified ultrasound echo signal, and the stimulation was not continuous in order to mimic the USVs emitted by rats. As reported in Figure 5, the A1 receptive fields were stimulated with processed echoes at 22 kHz (acousto-electric transduction (AET) at 22 kHz, AET-22 kHz) and at 50 kHz (AET-50 kHz). The electrical stimulation was different for the two kinds of vocalizations: a train of monophasic rectangular pulses of a 0.16 s duration (11 calls) for emulating USVs at 22 kHz and a train of monophasic rectangular pulses (26 calls) of 0.06 s for USV at 50 kHz. It operates with a maximum voltage of 2.5 V.

#### 2.2.3. Control Experiment with Acoustic Stimulation

In the control experiment, the rat was immobilized and placed in an anechoic chamber, while the US transducer was placed close to the external ear (at a distance of 10 cm) in order to verify the far-field condition. Spatial peak-temporal peak intensity was evaluated for both USVs using a wideband system composed by a conditioning amplifier (Bruel and Kjaer NEXUS 2692-C) and a ¼” free-field microphone (Bruel and Kjaer, Type 4939) to set the same pressure level. Control experiments were repeated on two different rats in order to investigate the evoked neural oscillations during the natural sensorial encoding progress at 22 kHz and 50 kHz using vocalizations according to those reported in Reference [21].

#### 2.2.4. ECoG Recording

Microelectrical stimulation was carried out at the University Magna Graecia of Catanzaro in conformity with national and international laws and policies (EU Directive 2010/63/EU for animal experiments, ARRIVE guidelines, and the Basel declaration, including the 3R concept). The experimental protocols were approved by the local ethical committee of the University of Catanzaro. Five male Long–Evans rats (Harlan Italy, Correzzana, Milan, Italy) were used in this study. The animals were kept under controlled environmental conditions (2 ± 2 °C; 60 ± 5% humidity; 12 h/12 h light/dark cycle; light on at 19.00). All efforts were made to minimize animal suffering and to keep the number of animals used to a minimum. Each animal was anesthetized through the administration of a mixture of tiletamine/zolazepam (1:1; Zoletil 100; 50 mg/kg i.p.; VIRBAC Srl, Milan, Italy) and subsequently inserted into a stereotaxic apparatus in order to carry out an accurate craniotomy. In the dura mater, small holes in the skull allowed the surgical implantation of intracranial electrodes. The Pt-Ir electrodes, used for stimulating and recording electrical activity in the brain, consisted of a thin copper wire of 7–8 mm in length with a diameter of 10 μm which were implanted into the fronto-parietal cortex and the inferior colliculus (see Table 1). The wires were sufficiently spaced to avoid capacitive coupling among them, and they were electrically insulated except for the tip. The animals were allowed at least 1 week of recovery and were handled twice a day. 

A Stellate Harmonie ECoG System (Montreal, QC, Canada) was used to record the electrophysiological activity with a non-cephalic point of reference. Unipolar ECoG placed over the fronto-parietal cortex and the inferior colliculus (IC) was used to evaluate the ascending and descending pathways, as reported in Table 1.

#### 2.2.5. Data Analysis 

All ECoG data were processed and analyzed using MATLAB^®^ (The MathWorks, Natick, MA, USA) and Fieldtrip Toolbox software (http://fieldtrip.fcdonders.nl) [28]. 

First, a notch filter was used to remove the 50 Hz noise. Then, the ECoG recordings affected by eye movement, blinking, and muscle movements were pre-processed by applying both a preliminary visual inspection and an artifact rejection routine with z-score thresholds in a Fieldtrip Toolbox environment. The neural recordings were divided into 500 ms epochs and were then baseline corrected. Stimulation artifacts were removed by applying principal component analysis: The signal was decomposed into uncorrelated components, and artifact components were identified and removed for signal reconstruction [29]. After pre-processing, a time–frequency analysis was applied to determine any dynamic changes in the cortical responses induced by the electrical stimulation. Spectral analyses of the ECoG data were carried out with Fieldtrip Toolbox using a multi-taper approach based on discrete prolate spheroidal sequences [28,29,30]. For each epoch, we evaluated the ECoG PSD between 0.5 and 100 Hz with a frequency resolution of 0.5 Hz (2 s using a Hamming tapered window). The peak frequency and PSD were performed at the following frequency bands: delta (1–4 Hz), theta (4–10 Hz), alpha (9–12 Hz), beta (12–25 Hz), and gamma (25–100 Hz).

Statistical analysis was performed with R 3.3.1 (http://www.r-project.org). Non-parametric tests were used to determine the significant differences between stimulation and post-stimulation epochs in order to verify the overall effects of electrical stimulation. Pearson’s chi-square test was applied for normal distribution, and the Kruskal–Wallis test was then fitted to perform the analysis of variance. Significance was determined at the *p* = 0.05 cutoff level.

## 3. Results

### 3.1. Identification of the A1 Receptive Fields

The A1 receptive fields were identified through the different neural responses. Raster plots and PSTHs were evaluated in order to identify an activity patterns of the primary auditory cortex during the presentation of the USVs. 

**Figure 6:**
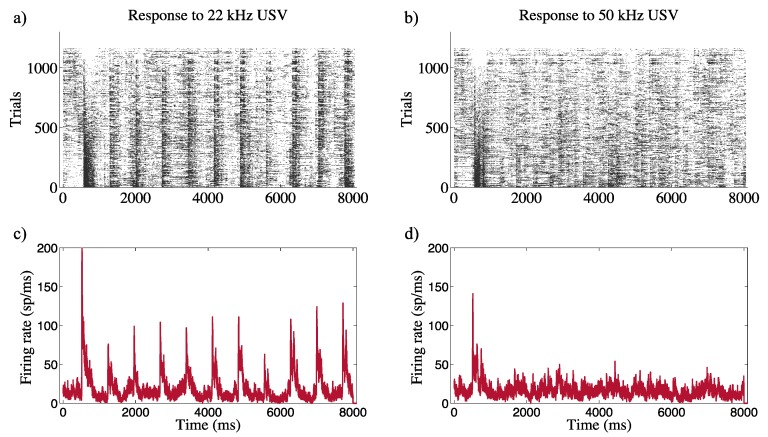
Raster plots and relative PSTHs for the multiunit activity of A1 to ultrasound at 22 kHz (**a**,**c**) and 50 kHz (**b**,**d**). The baseline was evaluated at 510 ms after USV onset; subsequently, an inter-stimulus interval (ISI) was used following the USV to avoid overlap during the 20 repetitions. The spectro-temporal characteristics of the neuronal discharge pattern were correlated with the acoustical structure of the vocalizations.

Elicited firing patterns were classified in transient (phasic) or sustained (tonic) responses to the stimulus onsets and/or offsets [21]. A tonic firing pattern was defined as a consistent, selective firing rate in response to the stimulus. Selectivity was observed during the intervals between calls. Neurons that initiated firing during the onset of stimulation were classified as belonging to the phasic response type. Phasic firing activity reflected only the early instantaneous tone-evoked response that decreased during the duration of the overall acoustic stimulation. Examples of tone-evoked responses are shown in Figure 6a–c and phasic excitation in Figure 6b–d. 

During the presentation of the 22 kHz USVs, the Al neurons followed the waveform of the stimulus, and spiking activity was typically selective for the 22 kHz subsets of USVs. Figure 6a–c highlights the sustained “tonic” responses for each burst in the stimulus train, with a peak every 550 ms from the onset of the overall train, whereas the response patterns to 50 kHz calls resulted in a neural pattern synchronized only to the onset of the incoming stimuli, assigning them to the phasic excitation category (Figure 6b–d).

**Figure 7:**
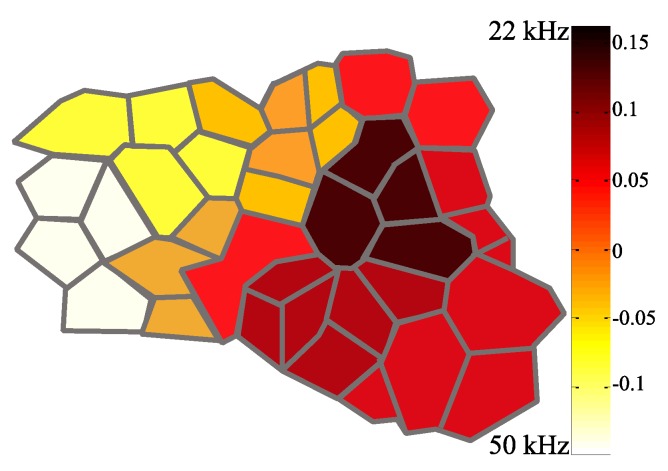
Example of an A1 map illustrating the sensitivity index (*SI*) of the recorded sites.

The A1 areas were selectively specialized in processing the two vocalizations which were identified and distinguished from each other through a comparison of evaluation mean responses and sensitivity indices. Each firing rate during a call presentation was compared with the baseline to show a cortical-specific activation. The distribution of the SI showed a stronger tone-evoked response occurring at the 50 kHz USV in the anterior direction relative to the stereotaxic coordinates. Presentation of the 22 kHz tone predominantly elicited response peaks in the posterior areas. As shown in Figure 7, the spatial relationship of the two USV responses was clearly distinct, resulting in a clear tonotopic gradient from the anterior-to-posterior representation of high-to-low frequencies. Each Voronoi tessellation showed the best frequency of neurons in reference to a specific channel: the 50 kHz areas are indicated in white in contrast to the 22 kHz zones shown in red.

**Figure 8:**
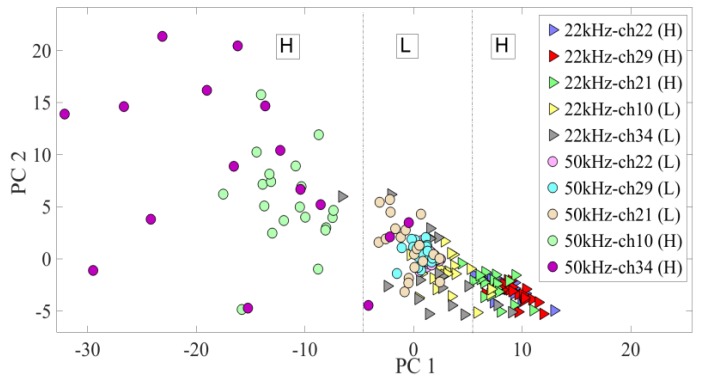
Functional grouping obtained with the PCA technique in response to USVs at 22 kHz and 50 kHz, each one presented 20 times. The PCA matrix was developed by taking into account 5 channels with the highest and lowest sensitivity indices (*SI*). H indicates the highest SI (channels 21, 22, and 29 for 22 kHz (triangles), and channels 10 and 34 for 50 kHz (circles)). Contrarily, L indicates the lowest SI (channels 10 and 34 for 22 kHz (triangles), and channels 21, 22, and 29 for 50 kHz (circles)). PCA classified the H band at the right and the left of the plot for the two different stimuli, in contrast to the L range at the center for both vocalizations.

An assessment of the two distinct regional functional properties was performed using PCA. The PCA plots for channels showing high (H) and low (L) sensitivity indices for the two chosen ultrasonic vocalizations can be seen in Figure 8. 

The PCA classified MUAs into the following three functional groups: the lowest spiking rate was localized in a central band, while the highest responses were localized in the right (22 kHz) and left (50 kHz) bands. During experimentation, the detection and classification of the two ethological vocalizations validated the previously calculated SI index.

The experimental results allowed the identification of potential stimulation sites through the evaluation of the tonotopic gradient of high-to-low best frequencies (BFs) oriented in the anterior-to-posterior direction in the A1.

### 3.2. Neural Modulation with the Biomimetic Sonar System

**Figure 9:**
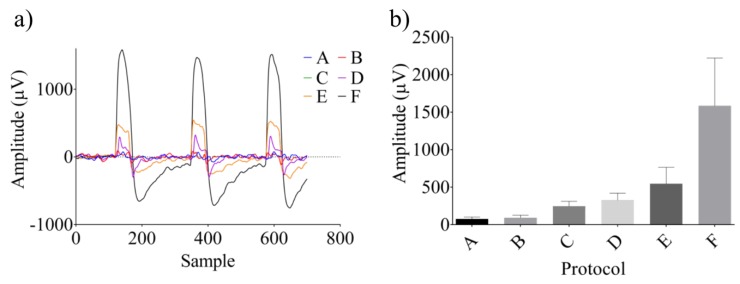
Resulting electrocorticography (ECoG) during electrical stimulation at AET-50 kHz with the same inter-stimulus interval (ISI) and number of trains (*n* = 10) and different increasing amplitudes according to the protocols from A to F; (**a**) the amplitude increases from 100 µV up to 8 times the baseline amplitude; (**b**) the ECoG traces are linearly dependent from Protocols B to F.

The ICMS by means of acousto-electric transduction of ultrasonic signals was applied into the receptive fields previously mapped in A1, while ECoG electrodes recorded the neural modulation in the frontal and parietal cortices and the inferior colliculus. The evoked responses are shown in Figure 9 as a function of stimulus amplitude. 

The cortical oscillations increased, starting at 100 µV up to 1500 µV (from Protocol A to Protocol F), and the resulting waveform showed a different morphology and a higher positive half-wave. The ECoG intensity displayed a linear dependence on the electric stimulus that increased, starting with the B protocol as shown in Figure 9b. Frequency-locking can be observed with the repetition rate synchronized with the rising and falling edge, whereas the increase of ISI did not influence the ECoG amplitude. 

**Figure 10:**
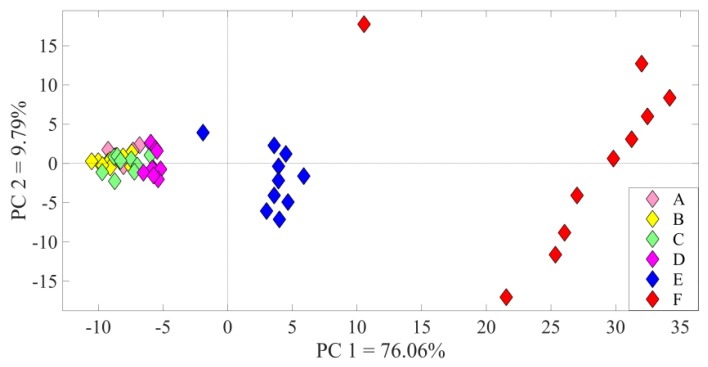
PCA was based on the covariance matrix of the observations. For each individual recording, PC1 and PC2 were kept, analyzed, and plotted. In the frontal cortex, each dot represents an ECoG epoch with an amplitude variation. The covariance matrix highlights a greater degree of dispersion with each increase in intensity. The legend refers to the Protocol Stimulation described in Figure 9.

**Figure 11:**
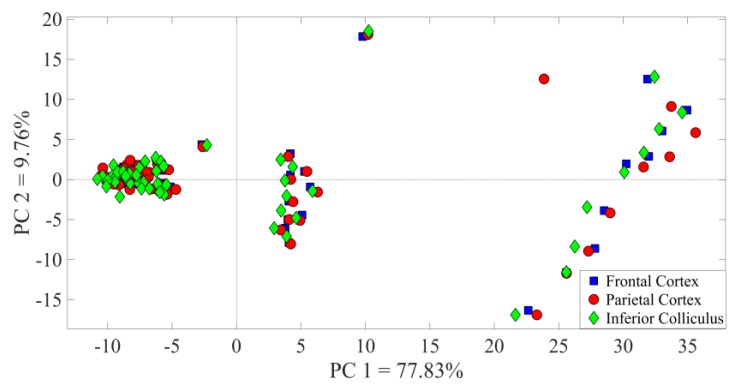
PCA applied in the frontal and parietal cortices and the inferior colliculus (ECoG traces). No significant differences can be seen in the score plot for the three neural areas.

The PCA was performed on the ECoG traces for a specified set of stimuli on *m* recordings of *n* samples (e.g., 6 epochs of the 3000 samples). The classification was performed for amplitude variations in the frontal and parietal cortices, as well as the inferior colliculus channels. Figure 10 shows that the first two principal components for a single channel captured 85.85% of the variance and gave insight into a greater dispersion of matrices with the increasing amplitude of stimulation. When comparing the three channels, no significant differences were observed (as shown in Figure 11).

**Figure 12:**
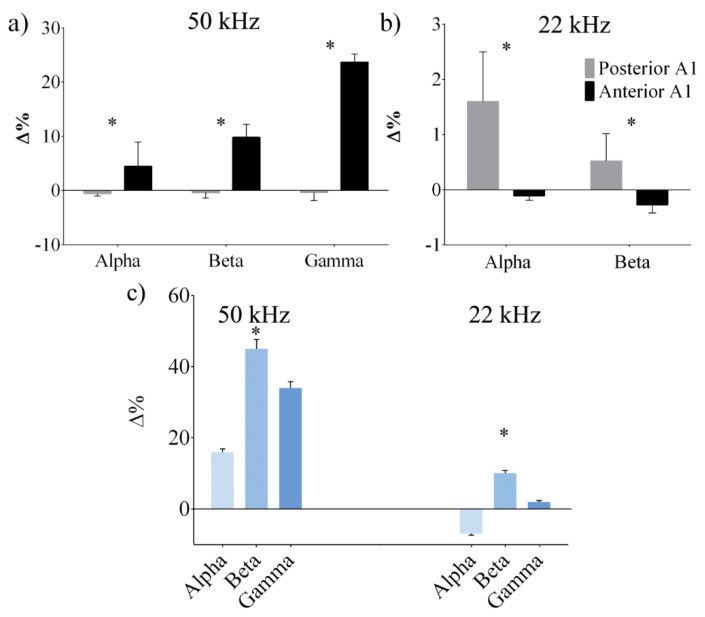
(**a**) Power spectral density (PSD) variations during electrical stimulation (AET-50 kHz) compared with the background. Alpha, beta, and gamma waves were activated in the anterior area that represents the tonotopical receptive field for ultrasound at 50 kHz in A1. The opposite zone was inhibited. (**b**) The graph shows the comparison of PSD versus background during stimulation with AET-22 kHz echoes properly transduced into electrical signals in the A1. The posterior area was activated in the alpha and beta content. (**c**) PSD variations during acoustical stimulation at 50 and 22 kHz compared with the background recorded in the frontal cortex. * *p* < 0.05.

The spectral analysis highlights the neuronal rhythms occurring at specific frequencies. Changes in power spectrum density indicated either the activation or deactivation of a specific region. Figure 12 shows how high-frequency alpha, beta, and gamma oscillations activated cortical areas during ICMS. Upon analyzing the posterior fields in the A1 that encoded for 22 kHz ultrasonic vocalizations, it can be seen that beta oscillations better contributed to processing alarm vocalizations because of the increase in PSD. This same area was selectively activated for the appropriate unipolar stimuli generated by AET-22 kHz. As shown in Figure 12b, the beta content increased when incoming stimuli matched the tonotopic frequency. Conversely, when different vocalizations occurred, the neural activity remained steady. Comparing the anterior area with the same stimulation protocol, it can be seen that the previous stimuli did not succeed in evoking responses, and the gamma waves increased in PSD content only for the UE-50 kHz as shown in Figure 12a. 

The alpha band contribution deserves deeper consideration. The PSD during stimulation and baseline was carried out when the anterior and posterior areas were electrically stimulated with the appropriate ultrasonic echoes. The PSD in the alpha band increased in the receptive fields, specifically in the posterior area, when stimulation was performed using 22 kHz echoes (see Figure 12b). On the contrary, inhibitory behavior was evidenced in the anterior area for 50 kHz stimulation (see Figure 12a). A control experiment was performed in two rats by evaluating cortical activity in response to external acoustic stimulation at 22 and 50 kHz. Figure 12c reports the PSD analysis of the evoked oscillations recorded in the frontal and parietal cortices, and near the cortico-fugal pathways of the inferior colliculus. Similar to the neural response induced by ICMS, in the control experiment, the evoked oscillations induced at 22 kHz show an increased PSD level in the beta band. Instead, neurons exhibit rhythmic spiking activity in the alpha, beta, and gamma range during 50 kHz acoustic stimulation. No significant differences were measured among the recording channels (Figure 12c).

## 4. Discussion 

In this study, we investigated the neuromodulation patterns that were induced by microelectrical stimulation produced through acousto-electric transduction of ultrasonic signals in adult Long–Evans rats. The experiments were aimed at exploiting the brain plasticity mechanism activated by a biomimetic sonar system in order to deliver environmental sensory information bypassing traditional afferent pathways. In a first step, these ultrasonic echoes were acoustically sent to characterize the neurophysiological response patterns of the A1 receptive fields. Subsequently, the corresponding echoes were transduced into unipolar electrical stimuli to neuromodulate cortical responses in the A1. In order to verify the effectiveness of biomimetic sonar compared with physiological ultrasound encoding, control experiments were performed, sending directly into the rat’s ear pure echoes at 22–50 kHz. The neural oscillations resulting from biomimetic sonar application in the A1 were compared with brain activity during the physiological ultrasound encoding in the frontal and parietal cortices and the inferior colliculus (control experiment).

Previous studies have provided consistent findings regarding the behavioral responses of rodents induced by ultrasound vocalizations [31,32,33]. In the present work, a correlation between neural activity and the acoustic features of the USVs was observed. Similar to Parsana [21], a discrimination of a tonic pattern at 22 kHz and of a phasic onset at 50 kHz were evaluated in the amygdala. However, we also extended the multidimensional scheme for classification of the firing patterns of A1 neurons. The A1 neurons were shown to fire in a time-locked manner, showing 11 peaks for each burst of incoming stimuli at 22 kHz with an ISI > 0.56 s, while during the highest number of calls (26 calls) and with an ISI < 0.20 s, the firing strength considerably decreased. Moreover, cortical spiking at 50 kHz was characterized by a phasic onset response consistent with an inhibitory effect [34]. The latter is often referred to as forward suppression, a specific sensory adaption in which the responses of the auditory neurons decrease over time as a consequence of faster stimuli [35]. This behavior was previously observed in rodents and echolocating bats with similar anatomical organization, in which USVs of distress are encoded by the A1 area with weak stimulus-locked responses after the first vocalization [36,37,38,39].

The spectral features of the two USVs were selectively represented by subsets of the cortical areas. All A1 neurons contributed to overall firing with a strength related to the specific tonotopic area in which it was situated, identified by the SI. The anterior area of the A1 showed a better response to higher frequency calls, while the posterior receptive fields were more strongly activated by lower frequency vocalizations. The neural discharge pattern reflected the tonotopic organization, confirming the findings of previous A1 mapping studies [40,41]. The spatially distributed neuron populations in the A1 cortical field were confirmed by the principal components of the neural spike data. The score plots highlighted two functional groups, clustering MUAS waveforms in response to ultrasound stimuli.

An intracortical microstimulation technique based on unipolar electrical stimuli generated by 22 kHz and 50 kHz echoes after acousto-electric transduction was investigated. During echolocation processes in the wild, mammals emit a variety of acoustic vocalizations, dynamically modifying ultrasound amplitude, in order to adapt them to their needs and environments [42]. When the auditory cortex was electrically stimulated by means of varying amplitudes, the ECoG and PSD amplitudes proportionally increased. The more intense ultrasound vocalizations covered a larger echolocation range and the brain dynamically adapted [42]. The incoming stimuli allowed a greater integration of cortical neurons [43]. In the score plot, PCA eigenfunctions showed an increased dispersion related to higher ultrasound amplitude, since more neural receptive areas contributed to firing. In addition, PCA was applied to cross-validate the neural response patterns of the frontal and parietal cortices and of the inferior colliculus. Neurophysiological studies on rats have indicated that the parietal cortex exhibits position-specific firing for navigation [44,45]. It receives multiple sensory inputs from visual, auditory, and somatosensory systems to produce egocentric, allocentric, and route-centric views—the three major frames of environmental references [44]. Working in parallel, the frontal cortex contributes to the achievement of high-level spatial representation for place recognition and path planning [46]. During experimentation, the last recording channel was inserted into the inferior colliculus, since it represents a relay station for all acoustic information [47]. Indeed, previous work evidenced that processing of vocalizations originates in the A1, and cortical representations of ultrasound waves are developed later in the inferior colliculus. This is because the A1 exerts a corticofugal influence in the descending pathway [19,48,49]. The PCA showed no significant differences among channels, highlighting the same processing phenomenon for the ascending and descending pathways. 

Spectral analysis evidenced that higher PSD levels in two different frequency bands (i.e., alpha and beta) were induced at AET-22 kHz in the posterior A1, whereas alpha and gamma oscillations were observed at AET-50 kHz in the anterior A1.

Previous studies have suggested that during different tasks, alpha activity is involved in an engagement or inhibition of specific cortical areas [44,50]. For instance, in task-specific populations, an increase in alpha oscillations is typically measured instead of a decrease in task-unrelated areas [51,52]. In response to the ultrasonic stimuli sent to specific A1 areas, the alpha waves provided a modulation of cortical excitability. 

Electrical stimulation of the posterior A1 at AET-22 kHz increased activity, whereas at AET-50 kHz a decrease of alpha oscillations was observed, reflecting functional inhibition. The opposite finding was observed in the anterior A1. The alpha waves modulated cortical excitability only during the activation of the specific populations devoted to encoding appropriate ultrasonic frequency, i.e., 50 kHz receptive areas, whereas disengagement was shown for 22 kHz echoes. 

Despite the different nature of the stimulation, the control experiment showed results in accordance with that observed using ICMS. Previous reports have shown an increase in beta oscillations in response to novel stimuli [53,54] during encoding and sensory information processing [55]. These findings are in good agreement with previous investigations in mustached bats, in which fast, rhythmic waves were recorded during both acoustic stimulation conditions [56] and social communication [57]. The results suggest that biomimetic ICMS triggers gamma oscillations in a similar way as a natural communication process [57].

## 5. Conclusions

Our primary aim was to verify whether a biomimetic sonar system emulating ultrasonic signals could induce neuromodulatory responses. In a way similar to echolocating mammals in nature, artificial biosonars can use ultrasound signals to detect obstacles by exploiting PVDF technology. We tested 22 kHz and 50 kHz echoes transduced into electrical signals to verify the neuromodulatory potential of directly stimulating the brain through the implantation of microelectrodes in the A1.

Ultrasound-based electric stimulation triggers high-frequency oscillations, encoding information about the stimulation sites and involving a large-scale integration in the brain. Spatial hearing experiments using ultrasound waves represent promising future avenues as stimuli useful for echolocation, modulating neural activity in the alpha, beta, and gamma bands.

## Figures and Tables

**Table 1:** Coordinates for recording and stimulating Electrode Implantation.

**Stimulating Electrodes**			
Left Auditory Cortex (anterior area: 50 kHz) AP = −3.3; L = 7.5; H = 4.7		Left Auditory Cortex (posterior area: 22 kHz) AP = −4.8; L = 7.2; H = 3.5	
**Recording Electrodes**			
Frontal Cortex AP = −2; L = 2.5	Parietal Cortex AP = −6; L = 2.5		Inferior Colliculus AP = −9.3; L = −1.5; H = 4.5
